# Metformin abrogates *Fusobacterium nucleatum*-induced chemoresistance in colorectal cancer by inhibiting miR-361-5p/sonic hedgehog signaling-regulated stemness

**DOI:** 10.1038/s41416-022-02044-6

**Published:** 2022-11-17

**Authors:** Xia-Lu Hong, Ta-Chung Yu, Xiao-Wen Huang, Ji-Lin Wang, Tian-Tian Sun, Ting-Ting Yan, Cheng-Bei Zhou, Hui-Min Chen, Wen-Yu Su, Wan Du, Hua Xiong

**Affiliations:** 1 Division of Gastroenterology and Hepatology; Shanghai Institute of Digestive Disease; NHC Key Laboratory of Digestive Diseases; State Key Laboratory for Oncogenes and Related Genes; Renji Hospital, School of Medicine, Shanghai Jiao Tong University, 145 Middle Shandong Road, Shanghai, 200001 China; 2 Division of Gastroenterology and Hepatology, Ningbo Hangzhou Bay Hospital, 1155 Second Binhai Road, Ningbo, 315336 China

**Keywords:** Cancer therapeutic resistance, Pathogens

## Abstract

**Background:**

Chemotherapy resistance is the major cause of recurrence in patients with colorectal cancer (CRC). A previous study found that *Fusobacterium (F.) nucleatum* promoted CRC chemoresistance. Additionally, metformin rescued *F. nucleatum*-induced tumorigenicity of CRC. Here, we aimed to investigate whether metformin could revert *F. nucleatum*-induced chemoresistance and explore the mechanism.

**Methods:**

The role of metformin in *F. nucleatum*-infected CRC cells was confirmed using cell counting kit 8 assays and CRC xenograft mice. Stemness was identified by tumorsphere formation. Bioinformatic analyses were used to explore the regulatory molecules involved in metformin and *F. nucleatum*-mediated regulation of the sonic hedgehog pathway.

**Results:**

We found that metformin abrogated *F. nucleatum*-promoted CRC resistance to chemotherapy. Furthermore, metformin attenuated *F. nucleatum*-stimulated stemness by inhibiting sonic hedgehog signaling. Mechanistically, metformin diminished sonic hedgehog signaling proteins by targeting the MYC/miR-361-5p cascade to reverse *F. nucleatum*-induced stemness, thereby rescuing *F. nucleatum*-triggered chemoresistance in CRC.

**Conclusions:**

Metformin acts on *F. nucleatum*-infected CRC via the MYC/miR-361-5p/sonic hedgehog pathway cascade, subsequently reversing stemness and abolishing *F. nucleatum*-triggered chemoresistance. Our results identified metformin intervention as a potential clinical treatment for patients with chemoresistant CRC with high amounts of *F. nucleatum*.

## Background

Colorectal cancer (CRC) incidence and mortality remain high, constituting a major public health burden [1]. Chemotherapy is the most common treatment to reduce tumor growth and inhibit tumor metastasis. 5-fluorouracil (5-Fu) and oxaliplatin are the first-line chemotherapeutic drugs in the treatment of patients with advanced CRC [2, 3]. However, CRC cells usually become resistant to chemotherapeutic agents, leading to tumor recurrence. It has been reported that more than 30% patients receiving surgery for stage II and III colon cancer are resistant to 5-Fu-based adjuvant chemotherapy within 8 years of follow-up [4]. Furthermore, about 50% of patients who were diagnosed with metastatic CRC were resistant to 5-Fu-based chemotherapy and their 5-year survival rate was lower than 15% [5]. Thus, it is necessary to determine the molecular mechanisms of CRC chemoresistance and develop new and effective strategies to improve chemosensitivity.

Chemoresistance of CRC is a complex process resulting from the interplay between intrinsic and extrinsic factors, including gene regulation, epigenetic modification, hypoxia, and the gut microbiota [6, 7]. Accumulating evidence indicates that the gut microbiota profoundly influences the effect of chemotherapy with three main outcomes: facilitation of drug efficacy; restraint and compromise of anticancer effects; and mediation of toxicity effects [8]. Some bacteria, such as *Lactobacillus johnsonii*, *L. murinus*, and *Bacteroidetes*, promote chemotherapy efficacy [9, 10]. However, some bacteria, such as *F. nucleatum*, a well-known CRC pathogenic factor, drive high chemoresistance [11–14]. Our previous work demonstrated that an increased abundance of *F. nucleatum* contributes to post-chemotherapy recurrence in patients with CRC [15]. As such, this is an important clinical consideration when evaluating conventional chemotherapeutic treatment of patients with CRC with a high amount of *F. nucleatum*. Therefore, a strategy capable of modulating the effects of *F. nucleatum* in the management of CRC is highly desirable. However, to date, the problem of CRC chemoresistance caused by *F. nucleatum* has not been solved. Our previous work pinpointed that metformin could elicit an antitumor effect by restoring the disordered intestinal microbiota and alleviating *F. nucleatum*-induced colorectal tumorigenesis [16]. Metformin, which has been used widely in type 2 diabetes (T2D) for more than 60 years, has attracted interest in the prevention and treatment of various cancers recently [17–20]. A phase II clinical trial reported a modest efficacy profile of a combination of metformin and 5-Fu in patients with refractory CRC [21]. These raised the possibility that metformin might play a role in treating chemoresistance in patients with CRC with high abundance of *F. nucleatum*. In the current work, we aimed to determine whether and how metformin affects *F. nucleatum*-induced CRC chemoresistance. We found that metformin plays a critical role in abrogating *F. nucleatum*-induced CRC chemoresistance in response to 5-Fu and oxaliplatin through the MYC/miR-361-5p cascade, and subsequently suppression of sonic hedgehog signaling and CRC cell stemness. Our approach might inspire a future combined treatment comprising metformin with chemotherapeutic drugs to managing recurrence in patients with CRC with high amounts of *F. nucleatum*.

## Methods

### Cell culture and treatment

Human CRC cell lines with different degrees of differentiation (HT-29, well differentiated; HCT 116, moderately differentiated) were purchased from the American Type Culture Collection (ATCC, Manassas, VA, USA) and cultured in McCoy’5 A medium (GIBCO, Carlsbad, CA, USA) with 10% (vol/vol) fetal bovine serum (FBS) (GIBCO) at 37 °C in a humidified 5% CO_2_ atmosphere. 1 mM metformin (Sigma-Aldrich, St. Louis, MO, USA) was used to treat CRC cells for 24–72 h. *F. nucleatum* at a multiplicity of infection (MOI) of 100 was used to treat CRC cells for 4 h. The miR-361-5p mimics and miR-361-5p inhibitor were transfected into CRC cells using the DharmaFECT transfection reagent (Thermo Fisher Scientific, Waltham, MA, USA). The lentivirus control and lentiviral vectors expressing miR-361-5p inhibitors were purchased from Shanghai OBiO medical biotechnology company (Shanghai, China).

### *F. nucleatum* culture

The *F. nucleatum* strain (ATCC 25586) was obtained from the ATCC. *F. nucleatum* was cultured for 24 h at 37 °C under anaerobic conditions in brain heart infusion (BHI) broth containing hemin, Vitamin K1, K_2_HPO_4_, and L-Cysteine.

### Mouse models

For the xenograft experiments, 4–5-week-old male *Balb/c* nude mice were purchased from the Shanghai Model Organisms Center (Shanghai, China) and housed in specific pathogen-free (SPF) conditions. HCT 116 cells (5 × 10^6^ or 2.5 × 10^6^) with different treatments were suspended in 100 μl of phosphate-buffered saline (PBS) and inoculated subcutaneously into the right axilla of the mice to establish the CRC xenograft model. Six days after subcutaneous injection, mice with xenograft tumors and no significant differences in tumor load were divided into different groups (*n* = 5). *F. nucleatum* (MOI = 100, 100 μl per mouse) were given by multipoint intratumoral injection, twice per week for 2 weeks. Metformin (125 mg/Kg) was administered via gavage every day for two weeks. Chemotherapeutic agents (FOX: 5-Fu 5 mg/Kg + Oxaliplatin 6 mg/Kg) were administered by intraperitoneal injection, twice per week for 2 weeks.

To explore the role of metformin in *F. nucleatum*-induced chemoresistance in vivo, we designed seven groups: (i) Control group; (ii) Metformin group; (iii) *F. nucleatum* group; (iv) FOX group; (v) FOX and metformin group; (vi) FOX and *F. nucleatum* group; (vii) FOX, *F. nucleatum*, and metformin group.

To explore whether metformin reversed *F. nucleatum*-mediated chemoresistance via miR-361-5p, we generated stable miR-361-5p knockdown HCT 116 cells by transducing lentiviruses expressing miR-361-5p inhibitors. We designed four groups: (i) FOX group; (ii) FOX and *F. nucleatum* group; (iii) FOX, *F. nucleatum* and metformin group; (iv) FOX, *F. nucleatum*, metformin, and miR-361-5p inhibitor group.

The length and width of the tumors were measured every 2–3 days. Tumor volume (mm^3^) was calculated as (length × width^2^) /2. If the tumor volume exceeded 2000 mm^3^, the mouse was killed for welfare reasons. After 2 weeks, the mice were killed humanely and subcutaneous tumors were collected for subsequent analysis of sonic hedgehog (SHH), GLI family zinc finger 1 (GLI1), SRY box transcription factor 2 (SOX2) and Nanog homeobox (NANOG) expression. Mouse experiments were conducted according to the guidelines approved by the Institutional Animal Care and Use Committee of Renji Hospital, School of Medicine, Shanghai Jiaotong University.

### RNA extraction and quantitative real-time PCR

Total RNA was extracted from HT-29 and HCT 116 cells using the Trizol reagent (Takara, Shiga, Japan). For the analysis of mRNA and primary microRNA (pri-miRNA), 1 μg of total RNA was reverse transcribed into first-strand cDNA using a PrimeScript RT Reagent Kit (Takara). For the miRNAs, 500 ng of total RNA was reverse transcribed using Mir-X TM miRNA First-Strand Synthesis Kit (Takara). The quantitative real-time PCR step was conducted using TB Green Premix Ex Taq™ II (Takara) on an ABI StepOnePlus Real-Time PCR System (Applied Biosystems, Foster City, CA, USA). The relative gene expression was calculated using 2^−ΔΔCt^ method and expressed as the fold change [22]. *ACTB* (encoding β-actin) and U6 were used, respectively, as endogenous references for the mRNA, pri-miRNA, and miRNA assays. The mRNA primers used in real-time PCR are shown in Table 1. The miRNA primers and pri-miRNA primers were purchased from GeneCopoeia (Rockville, MD, USA).

**Table 1:** List of the RT-PCR primers used in this study.

Primer name	Sequence (5’-3’)
GLI1 Forward	GGGTGCCGGAAGTCATACTC
GLI1 Reverse	GCTAGGATCTGTATAGCGTTTGG
SHH Forward	CAGTGGACATCACCACGTCT
SHH Reverse	CCGAGTTCTCTGCTTTCACC
SOX2 Forward	TGGACAGTTACGCGCACAT
SOX2 Reverse	CGAGTAGGACATGCTGTAGGT
NANOG Forward	ATAACCTTGGCTGCCGTCTC
NANOG Reverse	GATGCAGCAAATACGAGACCT
HES1 Forward	TGAGCACAGACCCAAGTGTG
HES1 Reverse	CCTCGGTATTAACGCCCTCG
TCF1 Forward	TGCACATGCAGCTATACCCAG
TCF1 Reverse	TGGTGGATTCTTGGTGCTTTTC
β-actin Forward	CTGGGCCTCGTCGCCCACATA
β-actin Reverse	CTGGGCCTCGTCGCCCACATA

### Western blotting

Proteins (60 μg) were separated using 8% or 12% sodium dodecyl sulfate polyacrylamide gel electrophoresis (SDS-PAGE), transferred onto polyvinylidene fluoride (PVDF) membranes (Bio-Rad, Hercules, CA, USA), and incubated with primary antibodies overnight. The membranes were then incubated with horseradish peroxidase (HRP)-conjugated secondary antibodies (1:3000, KangChen, Shanghai, China) and the signals were visualized using an enhanced luminescence (ECL) Kit (Thermo Fisher Scientific). The following commercial antibodies were used: anti-SHH (Cell Signaling Technology, Danvers, MA, USA), anti-GLI1 (Cell Signaling Technology), anti-SOX2(Cell Signaling Technology), anti-NANOG (Cell Signaling Technology), anti-MYC antibody (Abcam, Cambridge, UK) and anti-β-actin (Cell Signaling Technology). All antibodies were used at a dilution of 1:1000.

### Tumorsphere formation assay

CRC cells were exposed to *F. nucleatum* (MOI = 100) for 4 h. Then, the medium containing *F. nucleatum* was replaced with McCoy’5 A medium supplemented with 10% FBS. After 24 h, HT-29 and HCT 116 cells were seeded in low-adherent 96-well plates at a density of 400 cells per well in serum-free tumorsphere medium with or without 1 mM metformin. The tumorsphere medium consisted of Dulbecco’s Modified Eagle Medium/F12 (GIBCO) supplemented with necessary growth factors [23]. After a 4-day incubation, the tumorspheres were counted under a light microscope and their size were quantified using Image J software (NIH, Bethesda, MD, USA).

### Cell Counting Kit-8 (CCK-8) assay

CRC cells were seeded at 2500 cells per well into 96-well plates with 100 μl of medium. After attachment, HT-29 and HCT 116 cells were treated with different interventions for 72 h. Then, 10 μl of CCK-8 solution (Dojindo, kumamoto, Japan) was added to each well at specific times and the absorbance value at 450 nm was measured after 2 h of incubation.

### Data acquisition

For the analysis of cancer stem cell pathways, the data and sample information of GSE102573, GSE90944, and GSE67342 were downloaded from the Gene Expression Omnibus (GEO) (http://www.ncbi.nlm.nih.gov/geo/). GSE102573 comprises the expression profile of Caco-2 cells infected with *F. nucleatum* or not. GSE90944 comprises RNA sequencing (RNA-seq) data of HT-29 cells with or without *F. nucleatum* treatment. GSE67342 comprises expression data of LoVo cells treated by metformin. The single-sample gene set enrichment analysis (ssGSEA) score was calculated using Gene Set Variation Analysis (GSVA) in the R package with the chemical and genetic perturbations gene set collection (MSigDB C2 CGP; 3358 gene sets available) [24]. Differential expression analysis was conducted using the R package Limma and an adjusted (Bonferroni–Holm method) two-tailed *P* < 0.05 was considered statistically significant.

For the analysis of metformin-regulated genes, we used the dataset GSE67342, which includes gene expression of LoVo cells treated by metformin. An adjusted *P* < 0.1 was considered statistically significant.

### Dual luciferase assay

HT-29 and HCT 116 cells were co-transfected with 100 ng of the reporter plasmids and 10 ng of the pRL-TK-Renilla-luciferase plasmids using the FuGene transfection reagent (Promega, Madison, WI). At 6 h after transfection, the medium was replaced and cells were treated with metformin and/or *F. nucleatum*. At 72 h after transfection, dual luciferase activities were estimated using a Dual-Luciferase Assay Kit (Promega) according to the manufacturer’s instructions. Each transfection was performed in four complex wells and repeated twice.

### Statistical analysis

All data analyses were performed using GraphPad Prism version 7 (GraphPad Inc., La Jolla, CA, USA). Data are expressed as the mean ± standard deviation (SD) or the mean ± standard error of the mean (SEM). The differences between experimental groups were analyzed using an unpaired *t* test. In the figures, asterisks denote statistical significance (n.s., not significant, *P* > 0.05; *P < 0.05; **P < 0.01; ***P < 0.001; ****P < 0.0001).

## Results

### Metformin abolishes *F. nucleatum*-induced chemoresistance in vivo and in vitro

**Fig. 1:**
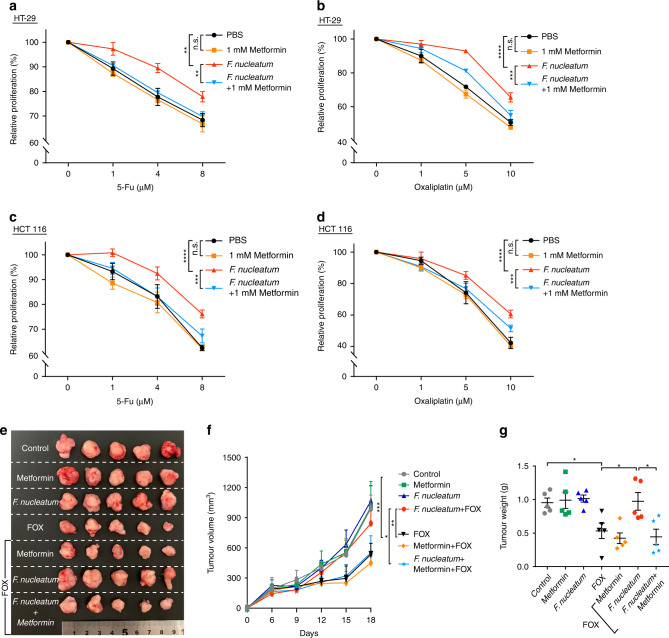
Metformin suppresses *F. nucleatum*-induced chemoresistance in vivo and in vitro. **a**, **b** Cell proliferation was detected by CCK-8 assays in HT-29 cells. HT-29 cells were co-cultured with *F. nucleatum* or treated with 1 mM metformin, and different concentration of 5-Fu (**a**) and Oxaliplatin (**b**); unpaired *t*-test. **c**, **d** Cell proliferation was detected by CCK-8 assays in HCT 116 cells. HCT 116 cells were co-cultured with *F. nucleatum* or treated with 1 mM metformin, and different concentration of 5-Fu (**c**) and Oxaliplatin (**d**); unpaired *t*-test. **e** Representative data of tumors in mice under different conditions. **f**, **g** Statistical analysis of tumor volumes (**f**) and weights (**g**) in the different groups, *n* = 5/group; values indicate mean ± standard error of the mean (SEM); unpaired *t*-test. n.s., *P* > 0.05; *P < 0.05; **P < 0.01; ***P < 0.001; ****P < 0.0001.

To explore the role of metformin in *F. nucleatum*-induced chemoresistance, we initially verified metformin’s effect on CRC cell proliferation. After treating CRC cells with different concentration of metformin, we observed that 500 μM and 1 mM metformin did not affect the proliferation of HT-29 cells (Fig. S1a) or HCT 116 cells (Fig. S1b). However, 5 mM and 10 mM metformin inhibited the proliferation in both CRC cell lines (Fig. S1a, b). Importantly, 1 mM metformin is approximately the physiological concentration in the colon of patients taking this drug [25]. In addition, our previous work demonstrated that an MOI of 100 of *F. nucleatum* had no effect on CRC cell proliferation but could trigger CRC cell chemoresistance [15]. Therefore, we chose 100 MOI of *F. nucleatum* and 1 mM metformin for subsequent experiments. HT-29 and HCT 116 cells were pre-infected with *F. nucleatum* for 4 h and then treated with a gradient concentration of 5-Fu or oxaliplatin in the presence of 1 mM metformin. In line with our previous observations, *F. nucleatum* decreased the cytotoxicity of 5-Fu and oxaliplatin in HT-29 cells (Fig. 1a, b) and HCT 116 cells (Fig. 1c, d), respectively, after 72 h. These results suggest that *F. nucleatum* promotes chemoresistance of CRC cells. Interestingly, metformin markedly enhanced the cytotoxicity of 5-Fu and oxaliplatin in *F. nucleatum*-infected HT-29 cells (Fig. 1a, b) and HCT 116 cells (Fig. 1c, d), but not in *F. nucleatum*-free HT-29 cells and HCT 116 cells. Thus, metformin could abolish *F. nucleatum*-induced CRC chemoresistance in vitro.

To further verify this conclusion, HCT 116 cells were inoculated into *Balb/c* nude mice, followed by treatment with metformin (125 mg/Kg/d), *F. nucleatum*, chemotherapeutic agents (FOX: 5-Fu 5 mg/Kg + Oxaliplatin 6 mg/Kg) and other manipulations. We observed comparable tumor growth (Fig. 1e), tumor volumes (Fig. 1f), and tumor weights (Fig. 1g) in the control, metformin, and *F. nucleatum* groups. As expected, tumor growth (Fig. 1e), tumor volumes (Fig. 1f), and tumor weights (Fig. 1g) were significantly decreased by FOX treatment, and this decrease was blocked by *F. nucleatum* infection. These data further demonstrated that *F. nucleatum* participated in CRC chemoresistance. Remarkably, *Balb/c* nude mice treated with *F. nucleatum* plus 1 mM metformin exhibited a better response to FOX, with impaired tumor growth, reduced tumor volumes, and lower tumor weights, when compared with the *F. nucleatum* plus FOX group (Fig. 1e–g). Collectively, these data supported the view that metformin reverses *F. nucleatum*-induced CRC chemoresistance.

### Metformin attenuates *F. nucleatum*-induced chemoresistance by inhibiting CRC cell stemness

**Fig. 2:**
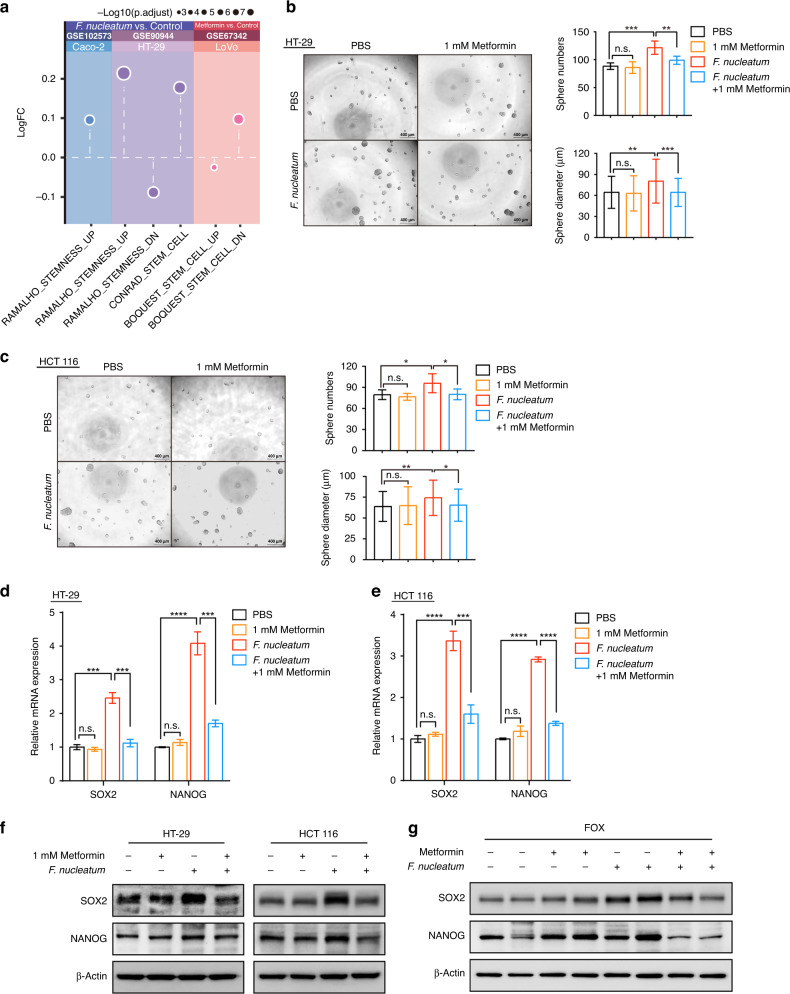
Metformin attenuates *F. nucleatum*-induced stemness in CRC cells. **a** ssGSEA analysis was conducted to show the alteration of stemness-related pathways in colorectal cancer cells treated with *F. nucleatum* or metformin. **b**, **c** Tumorspheres were observed by light microscopy in HT-29 (**b**) and HCT 116 (**c**) cells co-cultured with *F. nucleatum* or treated with 1 mM metformin. The number and size of tumorspheres was quantified in the right panel. Scale bar, 400 μm. **d**, **e** Real-time PCR was performed on stemness marker expression in HT-29 (**d**) and HCT 116 (**e**) cells co-cultured with *F. nucleatum* or treated with 1 mM metformin for 24 h; unpaired *t* test. **f** Western blotting was performed on stemness marker expression in HT-29 and HCT 116 cells co-cultured with *F. nucleatum* or treated with 1 mM metformin for 24 h. **g** Western blotting was performed to detect stemness-related proteins in xenograft tumors after different treatments. n.s., *P* > 0.05; *P < 0.05; **P < 0.01; ***P < 0.001; ****P < 0.0001.

We next explored the detailed mechanisms by which metformin abolished *F. nucleatum-*induced CRC chemoresistance. Given the crucial role of stemness in cancer chemoresistance [26], we hypothesized that metformin might rescue *F. nucleatum*-induced chemoresistance through inhibition of cancer stemness. To test this assumption, we performed bioinformatic analysis to predict the regulation of metformin and *F. nucleatum* in cancer stem cell pathways. We collected the gene expression data for CRC cells treated with metformin or *F. nucleatum* from GEO. Subsequently, ssGSEA showed that *F. nucleatum* upregulated cancer stem cell pathways, whereas metformin downregulated cancer stem cell pathways (Fig. 2a). Based on this data, we speculated that metformin might inhibit *F. nucleatum*-stimulated CRC stemness. Consistently, the tumorsphere formation assay showed that *F. nucleatum* increased the number and diameter of tumorspheres in both HT-29 cells (Fig. 2b) and HCT 116 cells (Fig. 2c). By contrast, metformin significantly inhibited the number and diameter of tumorspheres in *F. nuleatum*-stimulated HT-29 cells (Fig. 2b) and HCT 116 cells (Fig. 2c), but not in *F. nuleatum*-free cells.

In support of this data, we detected the expression of stemness markers after metformin and *F. nucleatum* treatment. NANOG and SOX2 are two important markers of stemness. *F. nucleatum* enhanced the mRNA and protein expression levels of SOX2 and NANOG in HT-29 and HCT 116 cells (Fig. 2d–f). Metformin abolished *F. nucleatum*-induced upregulation of SOX2 and NANOG at both the mRNA (Fig. 2d, e) and protein (Fig. 2f) levels. In line with the above results, western blotting analysis also showed that metformin downregulated *F. nucleatum*-stimulated SOX2 and NANOG expression in FOX-treated subcutaneous xenograft mice (Fig. 2g). Taken together, our data indicated that metformin attenuates *F. nucleatum*-induced chemoresistance by inhibiting CRC cell stemness.

### Metformin rescues *F. nucleatum*-induced stemness by inhibiting the sonic hedgehog pathway

**Fig. 3:**
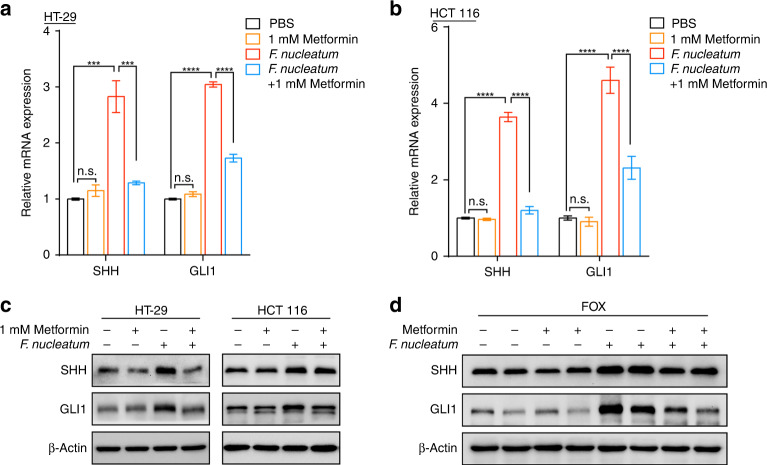
Metformin rescues *F. nucleatum*-induced stemness by inhibiting sonic hedgehog pathway. **a**, **b** Real-time PCR was performed to evaluate *SHH* and *GLI1* expression in HT-29 (**a**) and HCT 116 (**b**) cells co-cultured with *F. nucleatum* or treated with 1 mM metformin for 24 h; unpaired *t* test. **c** SHH and GLI1 levels were detected by western blotting in HT-29 and HCT 116 cells. The cells were co-cultured with *F. nucleatum* or treated with 1 mM metformin for 24 h. **d** Western blotting was performed to detect sonic hedgehog pathway-related proteins in xenograft tumors after different treatments. n.s., *P* > 0.05; ***P < 0.001; ****P < 0.0001.

We next examined how metformin rescued *F. nucleatum*-induced stemness in CRC cells. Notch, Wnt/β-catenin, and sonic hedgehog signaling pathways are known to play a decisive role in tumor stemness via regulating SOX2 and NANOG expression [27]. We hypothesized that Notch, Wnt/β-catenin, or sonic hedgehog signaling pathways were involved in the recovery effect of metformin in *F. nucleatum*-induced stemness. To test this hypothesis, we evaluated these three signaling pathways by testing the expression of critical transcription factors individually, including SHH (sonic hedgehog pathway), GLI1 (sonic hedgehog pathway), Transcription factor 1 (TCF1) (Wnt pathway), and Hes family BHLH transcription factor 1 (HES1) (Notch pathway). *F. nucleatum* enhanced the mRNA expression of *SHH*, *GLI1*, *TCF*, and *HES1* in HT-29 cells (Fig. 3a and Fig. S2a) and HCT 116 cells (Fig. 3b and Fig. S2b). This indicated *F. nucleatum* activates these three pathways. To our surprise, metformin reduced the mRNA expression of *SHH* and *GLI1* in *F. nucleatum-*infected HT-29 cells (Fig. 3a) and HCT 116 cells (Fig. 3b), but not in *F. nucleatum-*free CRC cells. Consistently, western blotting also showed metformin reduced SHH and GLI1 protein levels (Fig. 3c). By contrast, metformin could not downregulate the expression of *TCF1* and *HES1* in *F. nucleatum-*infected CRC cells (Fig. S2a, b). These data implied that the sonic hedgehog pathway might be involved in the metformin-mediated downregulation of *F. nucleatum*-induced stemness. In accordance with these results, CRC xenograft mice treated with FOX also showed that metformin diminished *F. nucleatum*-stimulated SHH and GLI1 protein levels (Fig. 3d). These data indicated that metformin inhibits *F. nucleatum*-induced stemness by inhibiting the sonic hedgehog pathway.

### MiR-361-5p inhibits the sonic hedgehog signaling pathway and stemness

Next, we conducted luciferase assays to investigate the molecular mechanism by which metformin and *F. nucleatum* control sonic hedgehog signaling. When the SHH ligand binds to Patched 1 (PTCH1) at the cell membrane, GLI1 is accumulated and activated. GLI1 is the final transcriptional effector and its expression reflects the activation of sonic hedgehog signaling [28]. Thus, we constructed the recombinant luciferase reporter plasmid pGL3-GLI1, containing the promoter region of *GLI1* ( − 979 to 33 nts) [29]. Luciferase assays showed that both *F. nucleatum* and metformin had no effect on the transcriptional activity of pGL3-GLI1 in HCT 116 cells (Fig. S3a). This result suggests that the regulation of GLI1 by metformin and *F. nucleatum* is not dependent on the direct transcriptional modulation of the *GLI1* promoter. Therefore, we sought to determine if metformin and *F. nucleatum* regulated the *GLI1* expression at the post-transcriptional level.

**Fig. 4:**
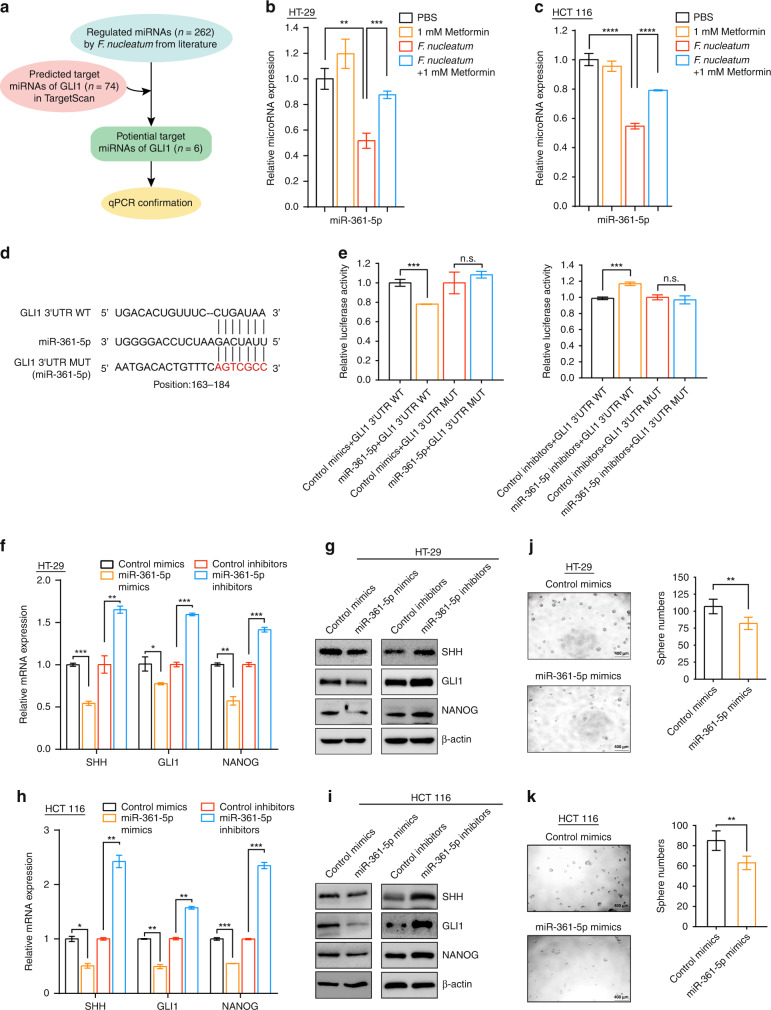
miR-361-5p inhibited the sonic hedgehog signaling pathway and stemness. **a** Schematic illustration of the target miRNA candidate screening process. **b**, **c** Expression of miR-361-5p was quantified by real-time PCR in HT-29 (**b**) and HCT 116 cells (**c**). The cells were co-cultured with *F. nucleatum* or treated with 1 mM metformin for 24 h; unpaired *t* test. **d** The predicted binding sequences for miR-361-5p within the human *GLI1* 3′ UTR. Seed sequences are highlighted. **e** Luciferase activity was measured in HCT 116 cells transfected with miR-316-5p mimics or control mimics, miR-316-5p inhibitors or control inhibitors for 72 h. The luciferase reporters expressing wild-type or mutant human *GLI1* 3′ UTRs were used; unpaired *t* test. **f**, **g** Real-time PCR (**f**) and western blotting (**g**) were performed in HT-29 cells to detect the expression levels of SHH, GLI1, and NANOG after transfection with miR-361-5p mimics or inhibitors for 48 h; unpaired *t* test. **h**, **i** Real-time PCR (**h**) and western blotting (**i**) were performed in HCT 116 cells to detect the expression levels of SHH, GLI1, and NANOG after transfection with miR-361-5p mimics or inhibitors for 48 h; unpaired *t* test. **j**, **k** Tumorspheres were observed by light microscopy in HT-29 (**j**) and HCT 116 (**k**) cells to detect the tumorsphere formation capability after transfection with miR-361-5p mimics; the number of the tumorspheres were quantified in the right panel. Scale bar, 400 μm; unpaired *t* test. n.s., *P* > 0.05; *P < 0.05; **P < 0.01; ***P < 0.001; ****P < 0.0001.

MicroRNAs (miRNAs) are important post-transcriptional regulators of gene expression [30]. We hypothesized that dysregulated miRNAs might be involved in metformin- and *F. nucleatum-*regulated *GLI1*. To test this hypothesis, we identified *F. nucleatum*-related miRNAs from two studies [15, 31]. *F. nucleatum* appeared to regulate 262 miRNAs significantly (Fig. 4a). Next, we used the bioinformatic tool, TargetScan (http://www.targetscan.org) to identify potential miRNAs that might regulate *GLI1*. Seventy-four potential *GLI1*-regulatory miRNAs were found (Fig. 4a). After overlapping these potential *GLI1*-regulatory miRNAs with the 262 *F. nucleatum*-related miRNAs, we identified six miRNAs (miR-361-5p, miR-509-3-5p, miR-509-5p, miR-103b, miR-4496, and miR-616-3p) that might regulate *GLI1* (Fig. 4a).

We validated whether these six miRNAs are regulated by metformin and *F. nucleatum* using real-time PCR. The results showed that 1 mM metformin alone had no regulatory effect on the six miRNAs (Fig. 4b, c and Fig. S3b, c). Interestingly, only miR-361-5p was significantly downregulated by *F. nucleatum* and this downregulation could be reverted by 1 mM metformin in both HT-29 (Fig. 4b) and HCT 116 cells (Fig. 4c). Although *F. nucleatum* inhibited miR-616-3p expression, the decreased miR-616-3p expression could not be rescued by 1 mM metformin in either CRC cell line (Fig. S3b, c). The other miRNAs were not significantly regulated by either *F. nucleatum* or metformin (Fig. S3b, c). Thus, these data implied that miR-361-5p might contribute to the downregulation of sonic hedgehog signaling in response to 1 mM metformin in *F. nucleatum*-infected CRC cells.

We next examined whether miR-361-5p could suppress sonic hedgehog signaling and stemness in CRC cells. Targetscan was used to predict the binding site of miR-361-5p in the 3′ untranslated region (UTR) of *GLI1*. Position163–184 of the *GLI1* 3′ UTR was identified (Fig. 4d). To verify whether the 3′ UTR of *GLI1* mRNA was a functional target of miR-361-5p, we performed dual luciferase reporter gene assays. The 3′ UTR sequences of *GLI1* (3′ UTR wild-type, WT) and the mutant 3′ UTR sequences of *GLI1* (3′ UTR mutant, MUT) were cloned into a luciferase reporter vector, separately (Fig. 4d). The dual luciferase assay demonstrated that miR-361-5p mimics suppressed the luciferase activity and miR-361-5p inhibitors elevated the luciferase activity in HCT 116 cells transfected with the WT *GLI1* reporter plasmid, but not with the MUT reporter plasmid (Fig. 4e). These findings indicated that *GLI1* is the specific target of miR-361-5p.

Then, to assess the function of miR-361-5p on the sonic hedgehog pathway and stemness, we transfected miR-361-5p mimics and inhibitors into HT-29 and HCT 116 cells, separately. Overexpression of miR-361-5p decreased the expression levels of SHH, GLI1, and NANOG at both the mRNA and protein level. By contrast, inhibition of miR-361-5p increased the expression of SHH, GLI1, and NANOG in HT-29 cells (Fig. 4f, g) and HCT 116 cells (Fig. 4h, i). In addition, the number and size of tumorspheres were reduced in the miR-361-5p overexpressing HT-29 (Fig. 4j and Fig. S3d) and HCT 116 cells (Fig. 4k and Fig. S3e). Thus, these data support the view that miR-361-5p suppresses the sonic hedgehog pathway and subsequently inhibits CRC cell stemness.

### Metformin reverts miR-361-5p in *F. nucleatum*-infected CRC cells via inhibiting MYC

**Fig. 5:**
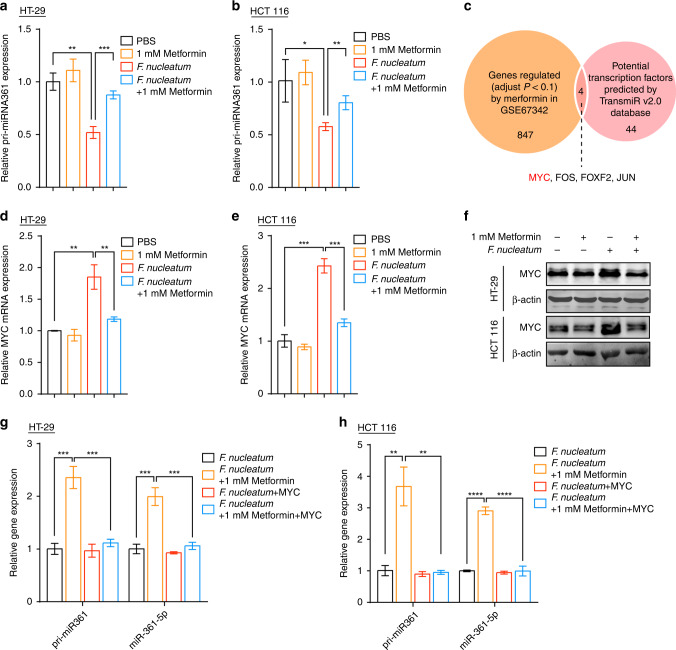
Metformin reverts miR-361-5p in *F. nucleatum*-infected CRC cells via inhibiting MYC. **a**, **b** Real-time PCR was performed in HT-29 cells (**a**) and HCT 116 (**b**) cells to detect the expression of pri-miR-361. The cells were co-cultured with *F. nucleatum* or treated with 1 mM metformin for 24 h; unpaired *t* test. **c** Venn diagram showing the candidate transcription factor screening process. **d**, **e** Real-time PCR was performed to evaluate *MYC* expression in HT-29 (**d**) and HCT 116 (**e**) cells treated with 1 mM metformin, *F. nucleatum*, and *F. nucleatum* plus 1 mM metformin for 24 h. **f** MYC levels were detected by western blotting in HT-29 and HCT 116 cells. The cells were co-cultured with *F. nucleatum* and/or treated with 1 mM metformin for 24 h. **g**, **h** Real-time PCR was performed to detect pri-miR-361 and miR-361-5p expression in HT-29 (**g**) and HCT 116 (**h**) cells co-cultured with *F. nucleatum* after MYC plasmid transfection, and subsequently treated with or without 1 mM metformin for 24 h. *P < 0.05; **P < 0.01; ***P < 0.001; ****P < 0.0001.

We next investigated the potential mechanisms responsible for the upregulation of miR-361-5p by metformin in *F. nucleatum*-infected cells. MiRNA genes are transcribed into primary miRNA (pri-miRNA) transcripts; therefore, we first detected pri-miR-361 expression using real-time PCR. *F. nucleatum* inhibited the expression of pri-miR-361 in HT-29 and HCT 116 cells (Fig. 5a, b). Metformin increased the expression of pri-miR-361 in *F. nucleatum*-infected HT-29 and HCT 116 cells, but not in *F. nucleatum*-free cells (Fig. 5a, b). These data indicated that metformin and *F. nucleatum* might regulate the transcription of miR-361-5p. To identify potential transcription factors of miR-361-5p, we employed the TransmiR v2.0 database (http://www.cuilab.cn/transmirwas) and predicted 48 transcription factors that might govern the expression of miR-361-5p directly (Fig. 5c). To explore metformin-regulated transcription factors, we analyzed microarray data to compare gene expression profiles of metformin-treated LoVo cells and control. After application of the filtering criterion (adjusted *P* < 0.1), a total of 560 downregulated genes and 292 upregulated genes were detected (Fig. 5c). Four transcription factors (*MYC*, *FOS*, *FOXF2*, and *JUN*) were identified after overlapping the 48 potential transcription factors and the metformin-regulated genes (Fig. 5c). Among these four transcription factor genes, *MYC* was the most significantly downregulated gene by metformin and was well characterized to bind to miRNA promoters directly [32].

To determine whether MYC contributed to the recovery effect of metformin in *F. nucleatum*-inhibited miR-361-5p, we first detected the expression of MYC in CRC cells treated with metformin and *F. nucleatum*. The levels of the MYC transcript (Fig. 5d, e) and protein (Fig. 5f) were enhanced in response to *F. nucleatum* infection in HT-29 cells and HCT 116 cells. Metformin reduced the levels of MYC mRNA (Fig. 5d, e) and protein (Fig. 5f) in *F. nucleatum-*infected HT-29 cells and HCT 116 cells, but not in *F. nucleatum-*free CRC cells. Moreover, metformin failed to upregulate pri-miR-361 and miR-361-5p when MYC was overexpressed in *F. nucleatum*-infected HT-29 cells (Fig. 5g) and HCT 116 cells (Fig. 5h). Collectively, the data suggested that metformin decreases miR-361-5p transcription in *F. nucleatum*-infected cells by inhibiting MYC.

### Metformin reverses *F. nucleatum*-induced stemness and chemoresistance via the miR-361-5p/ sonic hedgehog axis

**Fig. 6:**
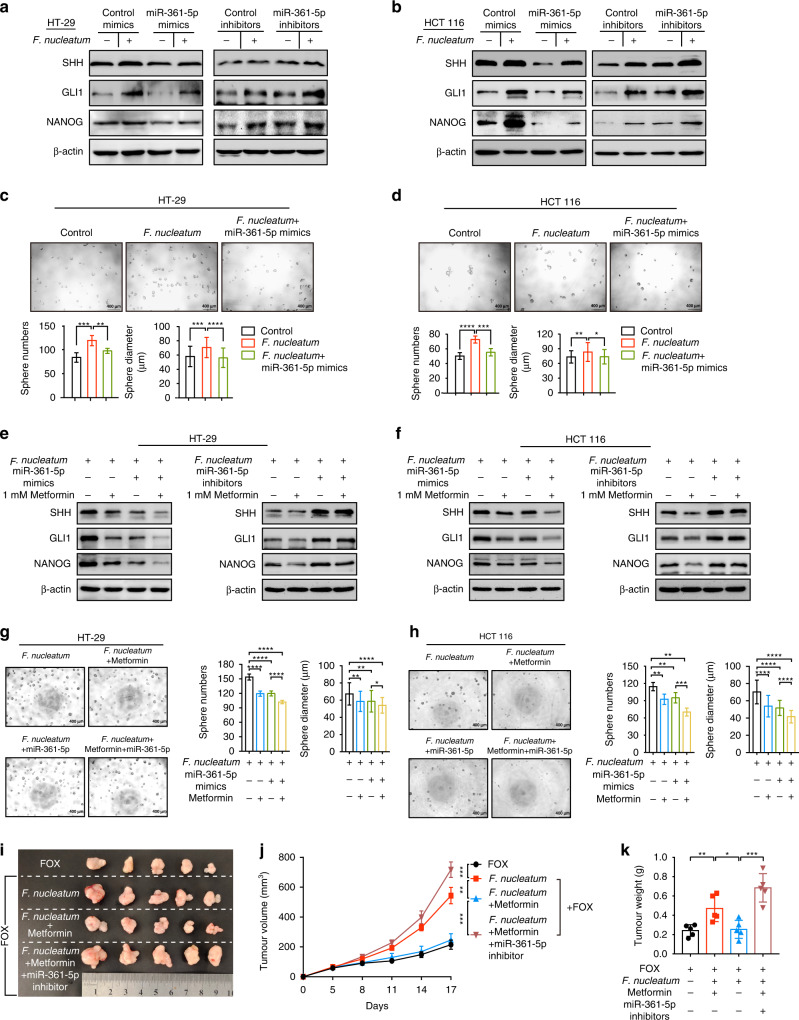
Metformin reverses *F. nucleatum*-induced stemness and chemoresistance via the miR-361-5p/sonic hedgehog axis. **a**, **b** HT-29 (**a**) and HCT 116 (**b**) cells were transfected with mimics or inhibitors of miR-361-5p. After culturing with *F. nucleatum* for 24 h, SHH, GLI1 and NANOG levels were detected by western blotting. **c**, **d** Tumorspheres were observed by light microscopy in HT-29 (**c**) and HCT 116 (**d**) cells transfected with miR-361-5p mimics, and then co-cultured with *F. nucleatum*. The number and size of the tumorspheres were quantified in the lower panel. Scale bar, 400 μm; unpaired *t* test. **e**, **f** Western blotting was performed to detect the levels of SHH, GLI1, and NANOG. After transfection with mimics or inhibitors of miR-361-5p, HT-29 (**e**) and HCT 116 (**f**) cells were treated with 1 mM metformin in the presence of *F. nucleatum* for 24 h. **g**, **h** After transfection with miR-361-5p mimics, HT-29 (**g**) and HCT 116 (**h**) cells were treated with 1 mM metformin in the presence of *F. nucleatum*. Tumorspheres were observed by light microscopy in HT-29 (**g**) and HCT 116 (**h**) cells. The number and size of the tumorspheres were quantified in the right panel. Scale bar, 400 μm; unpaired *t* test. **i** Representative data of the tumors in mice under different conditions. **j**, **k** Statistical analysis of tumor volumes (**j**) and weights (**k**) in the different groups, *n* = 5/group; unpaired *t* test. Values indicate mean ± standard error of the mean (SEM); unpaired *t*-test. *P < 0.05; **P < 0.01; ***P < 0.001; ****P < 0.0001.

To determine whether miR-361-5p regulates *F. nucleatum*-mediated sonic hedgehog pathway and CRC stemness, miR-361-5p mimics or inhibitors were transfected in *F. nucleatum*-treated CRC cells. Western blotting analysis revealed that miR-361-5p mimics decreased SHH, GLI1, and NANOG protein levels in HT-29 (Fig. 6a) and HCT 116 cells (Fig. 6b) cultured with *F. nucleatum*. Consistent with these results, a loss-of-function study showed miR-361-5p inhibitors increased the levels of SHH, GLI1, and NANOG in HT-29 (Fig. 6a) and HCT 116 cells (Fig. 6b), which presented the same effect as *F. nucleatum*. In addition, the number and size of tumorspheres stimulated by *F. nucleatum* were reduced in miR-361-5p overexpressing HT-29 cells (Fig. 6c) and HCT 116 cells (Fig. 6d). Thus, these data supported the view that *F. nucleatum* activates the sonic hedgehog signaling pathway and CRC stemness via the selective loss of miR-361-5p.

To address whether metformin blocked *F. nucleatum*-stimulated sonic hedgehog pathway and CRC stemness in a miR-361-5p-dependent manner, miR-361-5p mimics or 1 mM metformin were used to treat *F. nucleatum*-infected CRC cells. Similar to metformin, the miR-361-5p mimics decreased the SHH, GLI1, and NANOG levels stimulated by *F. nucleatum* in HT-29 cells (Fig. 6e) and HCT 116 cells (Fig. 6f). MiR-361-5p inhibitors abolished the inhibitory effects of metformin on SHH, GLI1, and NANOG levels in *F. nucleatum*-infected HT-29 cells (Fig. 6e) and HCT 116 cells (Fig. 6f). Consistently, tumorsphere formation assays showed that the miR-361-5p mimics repressed the number and size of tumorspheres in *F. nucleatum*-infected HT-29 cells (Fig. 6g) and HCT 116 cells (Fig. 6h). Besides, miR-361-5p inhibitors significantly blocked the inhibitory effects of metformin on tumorsphere numbers in *F. nucleatum*-stimulated cells (Fig. S3f).

To further assess whether metformin blocked *F. nucleatum*-stimulated chemoresistance in a miR-361-5p-dependent manner, we established stable miR-361-5p knockdown HCT 116 cells by transducing lentiviruses expressing miR-361-5p inhibitors. We then created subcutaneous tumor models via inoculation of the stable miR-361-5p knockdown HCT 116 cells into nude mice, followed by treatment with chemotherapeutic agents, *F. nucleatum*, and metformin. As expected, metformin reversed *F. nucleatum*-induced chemoresistance in control HCT 116 cells (Fig. 6i–k). However, the rescue effect of metformin in *F. nucleatum*-stimulated chemoresistance was abrogated by the miR-361-5p inhibitors in vivo, as shown by the lack of decrease in tumor growth (Fig. 6i), tumor volumes (Fig. 6j), and tumor weights (Fig. 6k). Altogether, we reasoned that metformin acts on *F. nucleatum*-infected CRC via the MYC/miR-361-5p cascade, which downregulates the sonic hedgehog signaling pathway, subsequently reversing CRC stemness and abolishing *F. nucleatum*-triggered chemoresistance (Fig. S4).

## Discussion

Chemotherapeutic agents, such as 5-Fu and oxaliplatin, remain the backbone of treatment for patients with CRC; however, the development of chemoresistance is the major cause for treatment failure. Cancer chemoresistance is a complex process and results from the interplay between intrinsic and extrinsic factors. Tumors with genetic and epigenetic alterations are critical for the CRC chemotherapeutic response [6]. Recent studies also showed that the gut microbiota controlled the response to chemotherapy by modulating the tumor microenvironment [33]. *F. nucleatum* abundance is associated with the properties of CRC, such as tumorigenesis, development, metastasis, and recurrence [34]. Through a combination of bioinformatic analyses, biological experiments, in vivo models, and clinical studies, we demonstrated that abnormal proliferation of *F. nucleatum* led to CRC chemoresistance and recurrence [15]. Given that broad spectrum antibiotics have a negative effect on the healthy intestinal microbiota, and no *F. nucleatum*-specific antimicrobial agent has been discovered, chemoresistance caused by *F. nucleatum* remains a thorny problem in the clinic [35]. Therefore, it is necessary to develop a safe and effective approach to eliminate chemoresistance caused by *F. nucleatum*.

Based on our previous work showing that metformin could attenuate *F. nucleatum*-induced tumorigenesis [16], we further explored the effect of metformin on *F. nucleatum*-induced chemoresistance. To our surprise, metformin abrogated *F. nucleatum*-induced CRC chemoresistance in CRC cells and xenograft mice. However, how metformin affects *F. nucleatum-*mediated chemoresistance was unknown. A distinct tumor cell subpopulation with stemness, known as the cancer stem cell population, exists in cancers, which mediates chemoresistance and metastatic progression [36, 37]. The use of cancer stemness inhibitors, such as napabucasin, can overcome chemoresistance in human cancers [38–40]. Emerging evidence supports the view that bacterial infections, such as *Enterococcus faecalis*, nonpathogenic *E. coli*, and Enterotoxigenic *Bacteroides fragilis*, might stimulate cancer cell stemness via upregulated expression of stemness markers [41–43]. Our bioinformatic studies demonstrated that a stemness-related pathway is enriched in the modulation of metformin and *F. nucleatum* in CRC cells. Indeed, the effect of metformin depends on the specific regulation of stemness in *F. nucleatum*-infected CRC cells. Accordingly, metformin decreased the expression of stemness-related proteins, SOX2 and NANOG, and inhibited tumorsphere formation in CRC cells co-cultured with *F. nucleatum*. Thus, we concluded that metformin attenuates *F. nucleatum*-induced chemoresistance by inhibiting stemness in CRC cells. These data might explain why metformin recovers chemosensitivity in *F. nucleatum*-infected CRC cells and xenograft mice.

We dissected the mechanisms by which metformin regulates *F. nucleatum*-induced stemness in CRC cells. Aberrant activity of the sonic hedgehog pathway has been linked to stem cell self-renewal and chemoresistance in variety of solid neoplasms [44]. We demonstrated that sonic hedgehog signaling is a bridge connecting metformin- and *F. nucleatum*-modulated stemness and chemoresistance. Metformin abolishes *F. nucleatum*-stimulated stemness and subsequently chemoresistance by selectively suppressing sonic hedgehog signaling. Furthermore, metformin and *F. nucleatum* do not affect the transcription of *GLI1*, which encodes the key effector of the sonic hedgehog pathway. Given that miRNAs are critical post transcriptional regulators [30], our bioinformatic and functional studies revealed miR-361-5p targets *GLI1* and inhibits sonic hedgehog signaling and stemness. In addition, metformin reverses *F. nucleatum*-stimulated stemness, sonic hedgehog signaling and chemoresistance in a miR-361-5p-dependent manner. In line with this notion, we found that metformin decreases miR-361-5p transcripts in *F. nucleatum*-infected cells by downregulating MYC expression. Our data suggested that metformin reverses *F. nucleatum*-induced stemness by inhibiting the MYC/miR-361-5p/sonic hedgehog signaling axis, and then biologically and mechanistically reverting CRC chemoresistance induced by *F. nucleatum*.

In addition to its biological importance, our work might be relevant in the clinical management of patients with CRC. The abundance of *F. nucleatum* is associated with the risk of CRC chemoresistance and recurrence; therefore, combining conventional chemotherapeutic regimens with metformin might be an effective strategy to reverse CRC chemoresistance in patients with high amounts of *F. nucleatum*. Our results highlighted the need for a clinical trial of metformin as a potential treatment in *Fusobacterium*-associated CRC chemoresistance. Furthermore, an important question raised by our data is whether metformin directly affects *F. nucleatum*. It has been reported that metformin exerts its hypoglycemic benefits partly through alteration of the abundance of certain members of the gut microbiota, such as *Bacteroides fragilis* and *Akkermansia muciniphila* [45–48]. Researchers also found that metformin could regulate microbial folate and methionine metabolism, which is required for bacterial growth [46, 49]. These provide the possibility that patients with CRC might benefit from the direct modulation of metformin on *F. nucleatum*. In addition to the details of the mechanisms by which metformin affects *F. nucleatum*-induced host responses, we will explore the direct effect of metformin on *F. nucleatum* colonization and dissemination in a future study.

Our results provide a foundation for the metformin–gut microbiota–host response network in CRC. This network regulation mode might be a direction for meaningful basic and translational medicine research in the future.

## Supplementary information

supplementary figure

## Data Availability

Previously published data sets were available in GEO under accession code GSE102573, GSE90944, and GSE67342.
