# Supplementary material for: Metformin abrogates Fusobacterium nucleatum-induced chemoresistance in colorectal cancer by inhibiting miR-361-5p/sonic hedgehog signaling-regulated stemness
Source: Br J Cancer. 2022 Nov 17;128(2):363–74. doi: 10.1038/s41416-022-02044-6 (PMC9902563; doi:10.1038/s41416-022-02044-6)
Supplement: Supplementary file 1 — supplementary figure [file 41416_2022_2044_MOESM1_ESM.doc]

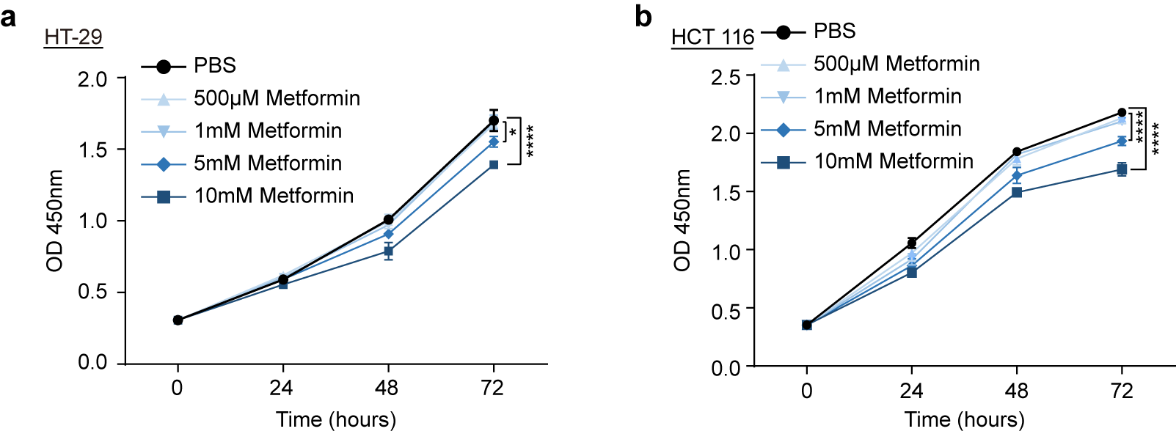


**Supplementary Fig. 1: The effect of metformin on CRC cell proliferation.**

**a, b** Cell proliferation was detected in HT-29 cells (a) and HCT 116 cells (b) treated with different concentrations of metformin for 72 hours*;* unpaired t test. *, *P* < 0.05; ****, *P* < 0.0001.


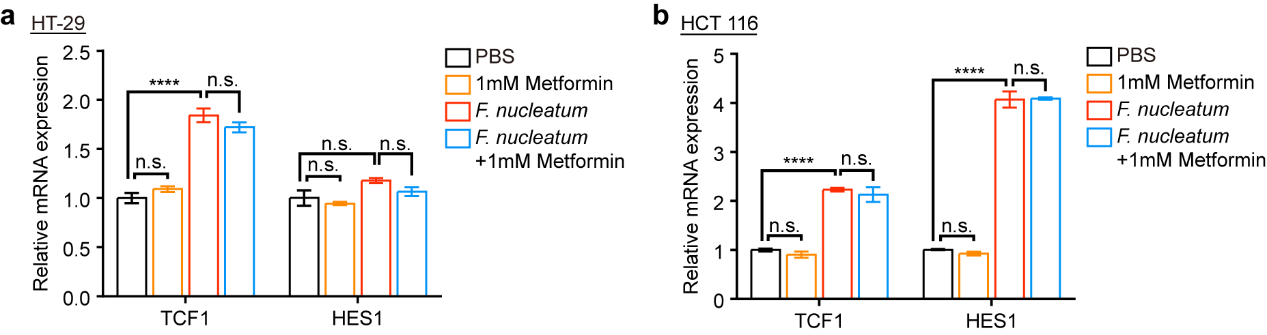


**Supplementary Fig. 2: The influence of 1 mM metformin and *F. nucleatum* on the Wnt and Notch pathways**

**a, b** Real-time PCR was performed to evaluate Wnt pathway (*TCF1*) and Notch pathway (*HES1*) expression in HT-29 (a) and HCT 116 (b) cells co-cultured with *F. nucleatum* or treated with 1 mM metformin for 24 hours; unpaired t test. n.s., *P* > 0.05; ****, *P* < 0.0001.


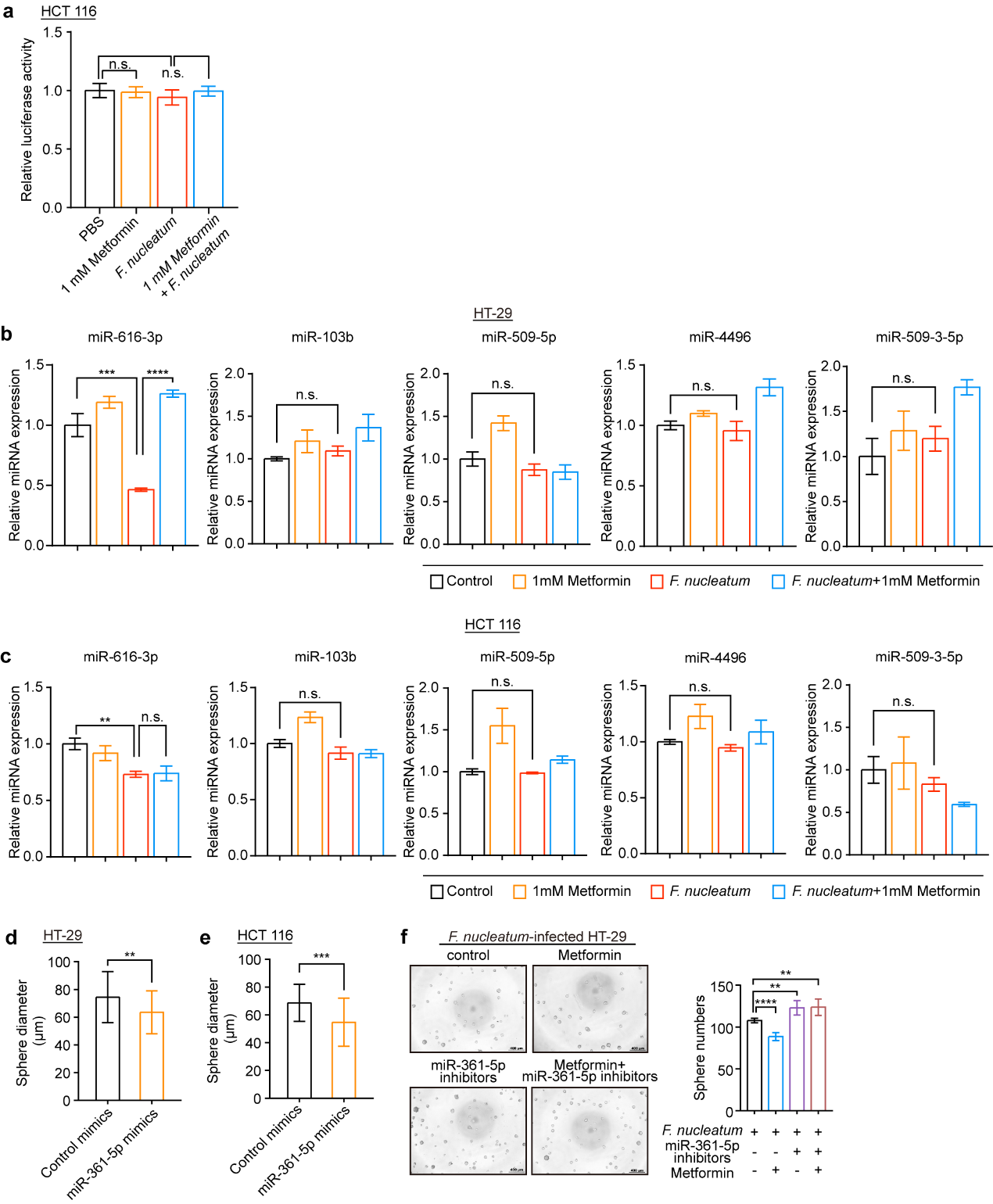


**Supplementary Fig. 3: The regulation of miRNAs by metformin and *F. nucleatum.***

**a** Luciferase assays were performed in HCT 116 cells. The cells were co-cultured with *F. nucleatum* or treated with 1 mM metformin after transfection with the *GLI1* promoter plasmids for 72 hours; unpaired t test. **b** Expression of candidate miRNAs (miR-103b, miR-509-5p, miR-4496, miR-616-3p and miR-509-3-5p) was quantified using real-time PCR in HT-29 cells. **c** Expression of candidate miRNAs (miR-103b, miR-509-5p, miR-4496, miR-616-3p and miR-509-3-5p) was quantified using real-time PCR in HCT 116 cells. **d, e** Tumorspheres were observed under a light microscope in HT-29 (d) and HCT 116 (e) cells to detect the tumorsphere formation capability after transfection with miR-361-5p mimics for 4 days; the size of tumorspheres were quantified. Scale bar, 400 μm, unpaired t test. **f** Tumorsphere formation was observed in HT-29 cells. The cells were transfected with miR-361-5p inhibitors, and subsequently treated with 1 mM metformin in the presence of *F. nucleatum* for 4 days. The number of tumorspheres were calculated in the right panel. Scale bar, 400μm; unpaired t test. n.s., *P* > 0.05; **, *P* < 0.01; ***, *P* < 0.001; ****, *P* < 0.0001.


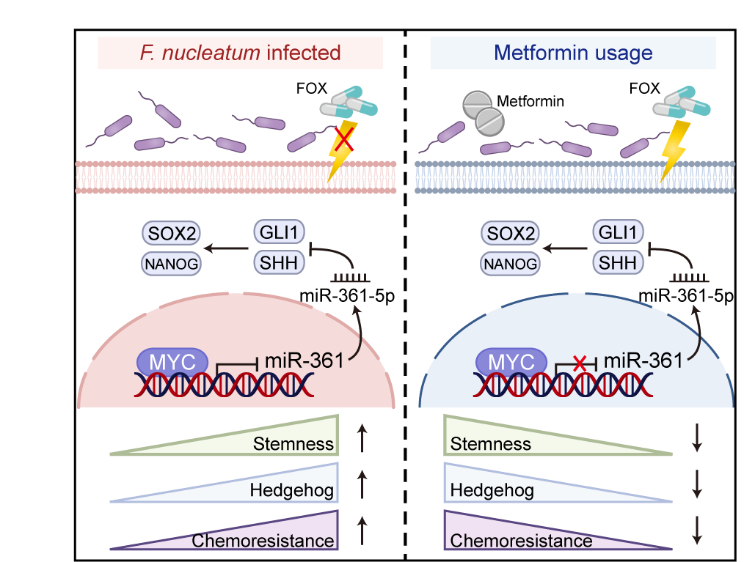


**Supplementary Fig. 4: Schematic model**

Metformin diminishes sonic hedgehog pathway proteins by targeting the MYC/miR-361-5p cascade to reverse *F. nucleatum*-induced stemness, thereby rescuing *F. nucleatum*‑induced chemoresistance in CRC.
